# Tissue-Specific *Salmonella* Typhimurium Gene Expression during Persistence in Pigs

**DOI:** 10.1371/journal.pone.0024120

**Published:** 2011-08-24

**Authors:** Alexander Van Parys, Filip Boyen, Bregje Leyman, Elin Verbrugghe, Freddy Haesebrouck, Frank Pasmans

**Affiliations:** Ghent University, Faculty of Veterinary Medicine, Department of Pathology, Bacteriology and Avian Diseases, Merelbeke, Belgium; Monash University, Australia

## Abstract

Salmonellosis caused by *Salmonella* Typhimurium is one of the most important bacterial zoonotic diseases. The bacterium persists in pigs resulting in asymptomatic ‘carrier pigs’, generating a major source for *Salmonella* contamination of pork. Until now, very little is known concerning the mechanisms used by *Salmonella* Typhimurium during persistence in pigs. Using *in vivo* expression technology (IVET), a promoter-trap method based on Δ*purA* attenuation of the parent strain, we identified 37 *Salmonella* Typhimurium genes that were expressed 3 weeks post oral inoculation in the tonsils, ileum and ileocaecal lymph nodes of pigs. Several genes were expressed in all three analyzed organs, while other genes were only expressed in one or two organs. Subsequently, the identified IVET transformants were pooled and reintroduced in pigs to detect tissue-specific gene expression patterns. We found that *efp* and *rpoZ* were specifically expressed in the ileocaecal lymph nodes during *Salmonella* peristence in pigs. Furthermore, we compared the persistence ability of substitution mutants for the IVET-identified genes *sifB* and *STM4067* to that of the wild type in a mixed infection model. The Δ*STM4067*::kanR was significantly attenuated in the ileum contents, caecum and caecum contents and faeces of pigs 3 weeks post inoculation, while deletion of the SPI-2 effector gene *sifB* did not affect *Salmonella* Typhimurium persistence. Although our list of identified genes is not exhaustive, we found that *efp* and *rpoZ* were specifically expressed in the ileocaecal lymph nodes of pigs and we identified *STM4067* as a factor involved in *Salmonella* persistence in pigs. To our knowledge, our study is the first to identify *Salmonella* Typhimurium genes expressed during persistence in pigs.

## Introduction

Non-typhoidal salmonellosis is one of the most important bacterial zoonotic diseases, yearly resulting in an estimated 155,000 deaths worldwide [1]. In European countries, *Salmonella enterica* subspecies *enterica* serovar Typhimurium (*Salmonella* Typhimurium) is the serovar most frequently isolated from slaughter pigs [2]. Porcine carcass contamination with *Salmonella* Typhimurium can largely be attributed to persistently infected pigs [3]. Transmission of *Salmonella* Typhimurium between pigs occurs mainly via the faecal-oral route. After ingestion by the pig, the bacterium will preferentially colonize its tonsils and ileum, where it adheres to the intestinal epithelium. This is followed by invasion and subsequent migration of the *Salmonella* bacterium to the underlying lymphoid tissues, like the ileocaecal lymph nodes, resulting in so called ‘carrier status pigs’ [4].

In the past, *Salmonella* interactions with hosts were largely examined in murine and avian models, while *Salmonella* Typhimurium behaviour in pigs is only poorly investigated. Due to the different and often hostile environments *Salmonella* must combat to successfully colonize one host, it is expected that the bacterium is equipped with a broad range of survival strategies, each one adapted to a certain biological niche. Therefore the bacterial genes involved in *Salmonella* survival in the tonsils might markedly differ from those essential for colonization of and persistence in for example the lymph nodes or the ileum in pigs [5].

The last decades, new techniques have been developed that allow high-throughput screening for genes important for microbial colonization and persistence in animal models [6], [7]. Recently, using signature-tagged mutagenesis (STM), *Salmonella* genes were identified that were specifically expressed in the deleterious gastric environment of pigs [8]. Furthermore, genes involved in *Salmonella* virulence in pigs shortly after oral inoculation have been identified [9], [10]. However, to date very little is known about the mechanisms employed by *Salmonella* Typhimurium during the persistent phase of infection of pigs. Because increasing evidence demonstrates that *Salmonella* Typhimurium behaves markedly different in various hosts, a thorough understanding of *Salmonella* pathogenesis in pigs is required to develop successful intervention strategies to cope with *Salmonella* infection of pigs.

Using the genome-wide approach of IVET, our goal was to identify genes specifically induced in porcine tonsils, ileum and ileocaecal lymph nodes at 3 weeks post oral inoculation as an indication of genes involved in *Salmonella* persistence in these 3 different organs of pigs.

## Materials and Methods

### Ethics statement

All animal experiments were approved by the ethical committee of the Faculty of Veterinary Medicine, Ghent University (EC2008/074; EC2009/021; EC2010/005 and EC2010/158 respectively).

### Bacterial strains and growth conditions

*Salmonella* Typhimurium strain 112910a phage type 120/ad, isolated from a pig stool sample, was used as the wild type strain (WT). A spontaneous nalidixic acid resistant derivative of the wild type strain was used in the mixed inoculation *in vivo* experiments. *Salmonella* Typhimurium substitution mutants Δ*purA*::kanR, Δ*sifB*::kanR and ΔS*TM4067*::kanR were constructed according to the one-step inactivation method described by [11] and slightly modified for use in *Salmonella* Typhimurium as described before [12]. Primers used to create the gene-specific linear PCR fragments are given as Supporting Information S1. For construction of the IVET pool, the Δ*purA* deletion mutant was obtained from the Δ*purA*::kanR substitution mutant, by excision of the kanamycin resistance cassette [11].

For oral inoculation of piglets, bacteria were cultured aerobically for 16 h at 37°C in 5 ml Luria–Bertani broth (LB; Sigma–Aldrich Chemie Gmbh, Steinheim, Germany) with the appropriate antibiotic(s), if required, included at the following concentrations: 20 µg/ml for nalidixic acid (Sigma–Aldrich); 100 µg/ml for kanamycin (Sigma-Aldrich) and 50 µg/ml for ampicillin (Sigma-Aldrich). The Δ*purA* strain was grown in LB enriched with 1.35% adenine and 0.337% thiamine. The bacterial 16 h cultures were then centrifuged at 2300 g for 10 min at 4°C and pellets were washed in Hank's buffered salt solution (HBSS; Gibco Life Technologies, Paisley, Scotland). After subsequent centrifugation at 2300 g for 10 min at 4°C, pellets were resuspended in 5 ml HBSS and diluted to the appropriate concentration for oral inoculation. The actual number of viable *Salmonella* colony forming units (CFU) per ml inoculum was determined by plating 10-fold dilutions on brilliant green agar (BGA; Lab M Limited, Lancashire, UK), supplemented with the appropriate antibiotic(s) for selective growth of the inoculated strain.

MacConkey agar (Oxoid, Basingstoke, United Kingdom) supplemented with 1% filter-sterilized lactose (Merck KGaA, Darmstadt, Germany) was used for growth of IVET transformants to assess their *lacZY* expression level.

### Construction of IVET pool

The pIVET1 plasmid is a derivate of the suicide vector pGP704 and contains a promoterless synthetic operon of *purA* coupled to *lacZY*, preceded by a *Bgl*II restriction enzyme site. This plasmid also possesses a mobilization site (*mob*), which results in wide bacterial host transfer through conjugation and an ampicillin resistance gene (*bla*). The IVET fusion pool was constructed as earlier described for *Salmonella enterica* serovar Enteritidis [13]. In short, *Salmonella* Typhimurium genomic DNA was purified and subsequently digested with the *Sau3AI* restriction enzyme (New England Biolabs, Ipswich, England), resulting in a library of 1–4 kb overlapping genomic DNA fragments. These fragments were then cloned in the *Bgl*II (New England Biolabs) site of the pIVET1 plasmid, upstream to promoterless wild type copies of *purA* and *lacZY*. The plasmids were transferred to *Escherichia coli* DH5αλpir by electroporation and approximately 100.000 different clones were pooled. pIVET1 fusion plasmids were then isolated using the Plasmid Midi Kit (Qiagen, Venlo, The Netherlands) and electroporated in the conjugative *Escherichia coli* SM10 λpir. Again, 100.000 different *E. coli* SM10 λpir clones were pooled. Fusion plasmids were then mobilized into the *Salmonella* Typhimurium Δ*purA* strain by conjugation using the *Escherichia coli* SM10 λpir as a donor strain. Integration of the pIVET1 plasmid in the chromosome resulted in a single cross over and therefore did not disrupt the wild type locus of the gene. In this way, *Salmonella* Typhimurium Δ*purA* transformants were obtained, in which the native promoter drove the *purA*-*lacZY* fusion, while the cloned promoter drove the expression of the wild type gene. Correct integration of the pIVET1 fusion plasmid in the genome was assessed by growth on MacConkey agar plates with 1% lactose, nalidixic acid, ampicillin, thiamine and adenine. The diversity of the IVET library was randomly tested by repeatedly purifying and subsequently sequencing colonies from MacConkey agar plates after conjugation between *E. coli* and Δ*purA Salmonella* Typhimurium. Approximately 15.000 different IVET transformants were purified and pooled, resulting in the final IVET bank that was stored at −70°C until further use.

### Promoter identification

The sequences that were cloned at the 5′ site of the *purA*-*lacZY* fusion were identified by a modification of the PCR-based method of [14]. Genomic DNA of IVET fusion strains of interest was isolated using the QIAamp DNA Mini Kit (Qiagen), and completely digested with the *Nla*III restriction enzyme (New England Biolabs). These DNA fragments were ligated to a ‘Y-linker’ using T4 DNA ligase (New England Biolabs). The linker sequences used to synthesize the ‘Y-linker’ are given as supplementary information. Prior to adding linker 1 to linker 2, the latter was phosphorylated at the 5′ end using T4 polynucleotide kinase (New England Biolabs). The mixture of both linkers was heated to 95°C and slowly cooled down to room temperature, to form the ‘Y-linker’. After ligation of the ‘Y-linker’ to the genomic DNA fragments, a PCR was performed using a *purA* primer, binding the *purA* sequence, and a primer binding the ‘Y-linker’ sequence (see supplementary information for primer sequences). The sequence between the *purA* and ‘Y-linker’ primer was thus sequenced. DNA sequences of IVET fusion strains that resulted in a band on gel electrophoresis were sequenced using the BigDye Terminator v3.1 Cycle Sequencing Kit (Applied Biosystems, CA 94404, USA) according to the manufacturer's guidelines. Sample sequences were determined using a 3100 Genetic Analyzer (Applied Biosystems). Identification of sequences of the cloned *Salmonella* promoters was done by BLAST analysis using the genome sequence of *Salmonella* Typhimurium LT2 (http://blast.ncbi.nlm.nih.gov/).

### *In vivo* experiments

Four-week-old piglets (commercially closed line based on Landrace) from a serologically *Salmonella* negative breeding herd (according to the Belgian *Salmonella* monitoring program) were used in the *in vivo* trials. The piglets arrived at the facility 5 days before they were inoculated. Prior to inoculation, the *Salmonella*-free status of the multiple piglets used in the *in vivo* experiments was tested serologically and bacteriologically. For the serological test, blood samples were collected from all piglets and serum was isolated and subsequently analyzed using a commercially available *Salmonella* antibody test kit (IDEXX, Hoofddorp, The Netherlands). Faeces were collected and enriched in tetrathionate broth overnight at 37°C. This suspension was then plated on BGA, incubated overnight at 37°C and visually checked for growth of salmonellae. Only piglets that were serologically negative and negative at faecal sampling were used. The animals were housed in separate isolation units at 25°C under natural day-night rhythm in HEPA-filtered stables, with ad libitum feed and water.

#### A. *Salmonella* Typhimurium Δ*purA* behaviour in piglets

To assess the suitability of a *Salmonella* Typhimurium *purA* mutant (Δ*purA*::kanR) for use in the current IVET approach, we first determined whether this mutant was significantly attenuated compared to the wild type. For this purpose, 6 *Salmonella*-free piglets were orally inoculated with a mixture of approximately 2×10^7^ CFU *Salmonella* Typhimurium wild type and 2×10^7^ CFU *Salmonella* Typhimurium Δ*purA*::kanR. Eight days post oral inoculation, the piglets were humanely euthanized and samples of the palatine tonsils, lymph nodes (ileocaecal, mesenteric and colonic), ileum and contents, colon and contents, caecum and contents, spleen, liver and faeces (1 sample per organ) were collected and the number of *Salmonella* bacteria was determined in each sample.

Prior to further processing, tissue samples were rinsed and cut to small pieces. Tissue and contents samples were then weighed and 10% (w/v) suspensions were made in buffered peptone water (BPW; Oxoid, Basingstoke, UK) after which the tissue material was homogenized with a Colworth stomacher 400 (Seward and House, London, UK). The homogenized samples were examined for the presence of bacteria by plating 10-fold dilutions on BGA with nalidixic acid or kanamycin for selective growth of the wild type *Salmonella* Typhimurium and the Δ*purA*::kanR substitution mutant respectively. BGA plates were incubated for 16 h at 37°C. When negative at direct plating, samples were pre-enriched for 16 h in BPW at 37°C and enriched for 16 h at 37°C in tetrathionate broth (Merck KGaA, Darmstadt, Germany) and then again plated on BGA with antibiotics. Samples that were negative after direct plating but positive after enrichment were presumed to contain 50 CFU *Salmonella* per gram tissue (detection limit for direct plating). Samples that remained negative after enrichment were presumed to contain 0 CFU *Salmonella* per gram tissue.

#### B. Screening for *in vivo* induced *Salmonella* Typhimurium genes in pigs

For IVET screening of *Salmonella* Typhimurium genes specifically induced during persistence in piglets, 8 four-week old *Salmonella*-free piglets were orally inoculated with approximately 2×10^8^ CFU of the *Salmonella* Typhimurium IVET pool. Three weeks post inoculation, all piglets were humanely euthanized and samples of the palatine tonsils, ileocaecal lymph nodes and ileum were collected and bacteriologically analyzed as described above. For monitoring the transcriptional activity of the isolated IVET transformants *in vitro*, tissue homogenates were plated on MacConkey lactose agar with ampicillin, nalidixic acid and adenine/thiamine.

This allowed detection of bacterial strains containing promoters expressed *in vivo* in the tonsils, lymph nodes and ileum (*purA* expression) and not *in vitro*. Fusion strains containing promoters induced *in vitro* showed red colonies (high-level *lacZY* expression) on MacConkey agar, whereas fusion strains carrying promoters inactive *in vitro* (low-level *lacZY* expression) displayed white to pink colonies on MacConkey medium. Because we were interested in genes that were specifically induced *in vivo* and not *in vitro*, approximately 500 colonies with low-level *in vitro lacZY* expression were picked up and purified prior to sequencing.

#### C. Reintroduction of identified IVET transformants in piglets to verify tissue-specific gene expression

To verify if the identified promoters from the initial IVET screening exhibited tissue-specific expression patterns, the 32 IVET transformants (corresponding to the 37 identified genes listed in Table 2) isolated and identified in the initial screening were pooled and reintroduced in 6 four-week old piglets. To assure that every single IVET transformant was equally present in the inoculum, each transformant was grown separately and in triplicate in 5 ml LB with the appropriate additives for 16 h at 37°C on a shaker and the optical density of each culture was then measured at a wavelength of 650 nm (OD_650_) using a microplate ELISA reader (Multiscan MS, Thermo Labsystems, Helsinki, Finland). One ml of each 16 h culture was pooled, washed in HBSS and the bacterial pellet was diluted to a concentration of 5×10^8^ CFU per ml HBSS. Subsequently, piglets were orally administered 2 ml of this inoculum, corresponding to approximately 1×10^9^ CFU of the IVET transformants pool. Each IVET transformant was thus theoretically represented by circa 3×10^7^ CFU. Twenty days post oral inoculation, all piglets were humanely euthanized and samples of tonsils, ileocaecal lymph nodes and ileum were collected. Tissue samples were processed as described above and plated on MacConkey agar with the appropriate additives. Plates were incubated overnight at 37°C. From each tonsil and lymph node sample, the cloned promoters in 24 IVET colonies were identified by sequencing as described above, except for the tonsil of 1 piglet and the lymph nodes of another piglet, from which only 18 and 20 colonies were sequenced, respectively. For the ileum samples, all isolated colonies were sequenced. If no IVET transformants were isolated from the ileum after direct plating, samples were pre-enriched and enriched as described above, and plated on MacConkey agar with the appropriate additives. Subsequently, 10 colonies per enriched ileum sample were sequenced.

#### D. Experimental inoculation of piglets with *Salmonella* Typhimurium Δ*sifB* and Δ*STM4067* strains

Following the IVET screening in pigs and based on the literature, 2 *in vivo* induced genes were selected of which substitution mutants were constructed. These mutants were then tested for attenuation compared to the wild type strain in a mixed infection experiment. For this *in vivo* trial, 14 piglets were randomly divided in 2 groups of 6 animals and 1 negative control group of 3 animals. Animals of the first two groups were orally inoculated with a mixture of approximately 2×10^7^ CFU *Salmonella* Typhimurium wild type and 2×10^7^ CFU *Salmonella* Typhimurium Δ*sifB*::kanR or Δ*STM4067*::kanR, respectively, while the negative control pigs were administered 2 ml PBS. Three weeks post oral inoculation, the piglets were humanely euthanized and samples of the palatine tonsils, ileum and contents, ileocaecal lymph nodes, caecum and contents and faeces were collected and bacteriologically analyzed as described above for the wild type/Δ*purA*::kanR mixed infection experiment.

### Statistical analysis

For the WT versus Δ*purA*::kanR, Δ*sifB*::kanR and Δ*STM4067*::kanR mixed infection experiments, a non-paramatric Mann-Whitney U test was performed to determine whether the log value of the WT/mutant ratios of the samples was significantly different from the log value of the WT/mutant ratios of the inocula before and after oral inoculation. The OD_650_ values of the 16 h cultures of IVET transformants were statistically evaluated using a one-way ANOVA. Tissue-specific gene expression patterns between tonsils and ileocaecal lymph nodes were analyzed for each gene using a McNemar's test with tonsils and lymph nodes as paired groups with two possible outcomes (expressed or not). Statistical analyses were performed with the SPSS Statistics 17.0 software (SPSS Inc., Chicago, USA). Differences with a *p*-value ≤0.05 were considered statistically significant; differences with a value 0.05<*p*≤0.1 were considered borderline significant.

## Results and Discussion

### The *Salmonella* Typhimurium Δ*purA*::kanR strain is severely attenuated in pigs

The wild type *Salmonella* Typhimurium was recovered from all organs and organ contents from all piglets, except for the ileum contents and faeces from 1 animal, while the *purA*::kanR substitution mutant was found in the tonsils of only 1 piglet and the faeces of another, eight days post oral inoculation (Table 1). The WT and Δ*purA*::kanR CFU isolated from the tonsils and the faeces were therefore not statistically different (*p* = 0.182 and *p* = 0.317 resp.). However, in all other analyzed samples the number of WT bacteria significantly differed from the number of Δ*purA*::kanR (*p* = 0.046 for each sample), except for the ileum contents in which the difference between both strains was statistically borderline (*p* = 0.096). These data clearly indicate that *purA* expression is required for *Salmonella* Typhimurium survival and persistence in pigs. This finding allowed us to use the pIVET1 system based on *purA* attenuation for the identification of *Salmonella* genes expressed during persistence in piglets.

**Table 1:** *Salmonella* Typhimurium wild type and Δ*purA*::kanR colonization of pigs.

Sample	Wild type		Δ*purA*::kanR	
	Frequency	Average log^10^ (CFU)±SD	Frequency	Average log^10^ (CFU)±SD
Tonsil	6/6	3.65±1,52	1/6	0.77±1.89
Ileocaecal lnn*	6/6	3.66±0.35	0/6	0
Mesenterial lnn*	6/6	3.78±0.28	0/6	0
Colonic lnn*	6/6	3.86±0.16	0/6	0
Ileum*	6/6	4.13±0.69	0/6	0
Ileum contents	5/6	2.92±1.63	0/6	0
Colon*	6/6	3.93±0.27	0/6	0
Colon contents*	6/6	4.21±0.46	0/6	0
Caecum*	6/6	4.22±0.48	0/6	0
Caecum contents*	6/6	3.99±0.04	0/6	0
Liver*	6/6	3.38±0.31	0/6	0
Spleen*	6/6	3.50±0.21	0/6	0
Faeces	5/6	2.62±1.88	1/6	0.33±0.82

Colonization of *Salmonella* Typhimurium WT and Δ*purA*::kanR strains in 6 piglets 8 days post oral inoculation with 2×10^7^ colony forming units (CFU) of both strains. The log^10^ CFU of the wild type and Δ*purA* per gram sample is given as the mean ± standard deviation (SD). The frequency shows the fraction of positive samples in relation to the total number of tissue samples (n = 6). An asterisk (*) indicates a statistically significant difference (p≤0.05) between the log^10^(WT/Δ*purA*::kanR) in the inoculum and the log^10^(WT/Δ*purA*::kanR) in the respective sample.

### IVET screening for *in vivo* induced genes

To screen for *Salmonella* Typhimurium genes that are specifically induced in porcine tissues during the persistent phase of infection, we constructed an IVET transformants pool covering the major part of the *Salmonella* Typhimurium genome [14]. We purified and subsequently sequenced 394 colonies from the tonsils, ileum and ileocaecal lymph nodes resulting in the identification of 37 different *Salmonella* Typhimurium genes that were induced 3 weeks post oral inoculation of piglets with the IVET pool and that might be essential for *Salmonella* persistence in pigs. These genes encode proteins that belong to diverse functional groups. Of these genes, 5 genes were identified in all 3 analyzed tissues. Furthermore, respectively 7, 4 and 12 genes were identified in the tonsils, ileum and ileocaecal lymph nodes only. Finally, several genes were identified from 2 different organs. Six cloned fragments contained 2 different genes. Since it can not be determined which gene(s) is (are) expressed in these fragments, the genes are discussed separately in the paragraphs below. The majority of the genes was expressed in only 1 piglet. This might be a consequence of the small number of piglets (n = 8) that was used in the IVET study, while we screened approximately 15.000 different IVET transformants. It is possible that after inoculation not all transformants had an equal chance to successfully infect piglets, creating a bottleneck. An overview of the identified genes, categorized according to their function and the tissue(s) in which they were expressed, is given in Table 2.

**Table 2:** List of *in vivo* expressed *Salmonella* Typhimurium genes in porcine tissues.

	Organ			
Gene function	Tonsil	Ileum	Ileocaecal lymph nodes	Gene product description*
Non-SPI encoded virulence factor	*sifB*		*sifB*	Secreted SPI-2 effector protein
Chaperone	*htpG* (2)	*htpG*	*htpG*	Heat shock protein 90
			*dnaK* (3)	Molecular chaperone
		*mopA*		Chaperone hsp60
		*cbpA*		Curved DNA binding protein
LPS biosynthesis			*rfaE*	Bifunctional heptose 7-phosphate kinase/heptose 1-phosphate adenyltransferase
Amino acid biosynthesis			*aroK*	Shikimate kinase 1
Protein biosynthesis	*efp*		*efp* (2)	Elongation factor P
	*rnt*	*rnt*	*rnt*	Ribonuclease T
	*rpsU*			30S ribosomal protein S21
			*rpoZ*	RNA polymerase subunit omega
			*rpoN*	RNA polymerase sigma-54 factor
Surface structure	*fljB*			Flagellin type 1
	*fliC*			Flagellin type 2
OMP assembly			*yaeT*	Outer membrane protein assembly factor
			*asmA*	Putative assembly protein
DNA replication	*dnaC* (2)	*dnaC*	*dnaC* (2)	DNA replication protein
	*dnaT* (2)	*dnaT*	*dnaT* (2)	Primosomal protein DnaI
	*gyrB*		*gyrB*	DNA gyrase subunit B
DNA repair			*nrdB*	Ribonucleoside-diphosphate reductase 1 subunit bèta
Anaerobic growth			*fnr*	Fumarate/nitrate reduction transcriptional regulator
	*pflC*		*pflC*	pyruvate formate lyase activase II
			*pflD*	putative formate acetyltransferase 2
Quorum sensing	*ydeW ( = lsrR)*			Putative transcriptional repressor
Metabolism			*yadF*	Carbonic anhydrase
	*artP*			Arginine transport system
			*yhbG ( = lptB)*	Lipopolysaccharide transport protein
	*kdgK*			Ketodeoxygluconokinase
	*lysS*			Lysyl-tRNA-synthetase
			*citG2*	Triphosphoribosyl-dephospho-CoA synthase
		*scsA*		Suppressor of copper sensitivity
Putative	*ybjP*			Putative lipoprotein
	*yggE*			Putative periplasmic immunogenic protein
		*STM3020*		Putative LysR family transcriptional regulator
	*STM4067* (3)	*STM4067*		Putative ADP-ribosylglycohydrolase
	*ygfA*	*ygfA*	*ygfA* (2)	Putative ligase
			*citX2*	Putative cytoplasmic protein

List of genes induced in the tonsils, ileum and ileocaecal lymph nodes of 8 pigs 3 weeks post oral inoculation with 2×10^8^ CFU of the *Salmonella* Typhimurium IVET library. If a gene was expressed in a certain organ of more than 1 piglet, the actual number of piglets is indicated between brackets. *Gene product descriptions according to the National Center for Biotechnology Information (http://www.ncbi.nlm.nih.gov/nuccore/NC_003197). SPI: *Salmonella* pathogenicity island; LPS: lipopolysaccharide.

### Chaperones

Four IVET identified genes encode chaperones or heat-shock proteins (hsp's) that guide correct folding of newly synthesized proteins and that protect the cellular components under stressful conditions. The *htpG* gene is the only chaperone encoding gene that was expressed in tonsils, ileum and ileocaecal lymph nodes. HtpG is an hsp90 homologue that is more than 2-fold expressed in *Salmonella* Typhimurium in response to the bactericidal/permeability increasing protein (BPI) from human neutrophils [15]. It is possible that the expression of *htpG* is induced in *Salmonella* Typhimurium following exposure to antimicrobial peptides present in the tonsils, ileum and/or ileocaecal lymph nodes of pigs. Such potential candidate is the palate, lung and nasal epithelium clone or SPLUNC1, a BPI-homologue secreted by the epithelial cells of the nasal cavity and the respiratory tract in pigs, although this protein has not yet been detected in porcine tonsils [16]. *dnaK* was expressed in the lymph nodes of 3 piglets and its gene product forms a chaperone machinery with co-chaperones DnaJ and GrpE, that protect from low pH and heat stress by binding non-specifically to unfolded polypeptides to produce or restore functional proteins. This gene was shown to be involved in the acid stress response of *Salmonella* Typhimurium in the gastric environment of pigs [8].

Two other chaperones were identified from the tonsils. MopA is involved in ceftriaxone resistance in *Salmonella* Typhimurium, is similar to the *E. coli* chaperonin GroEL and is transcribed extensively during heat recovery in *Salmonella* Enteritidis [17]. *cpbA* encodes a ‘curved DNA binding protein’, a molecular hsp40 chaperone, homologous to DnaJ and involved in bacterial responses to environmental stress. The expression of both genes is regulated by the virulence-associated bacterial-transcriptional regulator SlyA, involved in systemic but not enteric salmonellosis, resistance of *Salmonella* Typhimurium to oxidative stress and destruction of M cells in mice [18].

### LPS, amino acid and protein biosynthesis

Several genes were identified that play a role in biosynthesis pathways. RfaE encodes the gene for ADP-L-glycero-D-manno-heptose, a component of the *Salmonella* Typhimurium lipopolysaccharide (LPS) inner-core. A Δ*rfaE Salmonella* Typhimurium has an incomplete LPS and exhibits a lower growth rate, higher temperature sensitivity and decreased invasiveness of epithelial cells [19]. *Salmonella* Typhimurium strains defective in LPS are less resistant to the antimicrobials baicalin and novobiocin [20], rendering it possible that *rfaE* is expressed in the ileocaecal lymph nodes by *Salmonella* Typhimurium as a response to certain host antimicrobial proteins. AroK catalyzes the production of shikimate-3-phosphate from shikimate in the aromatic amino acid biosynthesis pathway. In *E. coli*, AroK is involved in resistance to the extended-spectrum penicillin mecillinam [21] and might play a role in the response of *Salmonella* Typhimurium to (yet unknown) porcine antimicrobial peptides.

The among bacteria highly conserved elongation factor P (encoded by *efp*) was identified in the tonsil and ileocaecal lymph nodes. Efp is homologous to the eukaryotic translation initiation factor eIF5A and is essential for protein synthesis and for growth and viability in *E. coli* [22]. The exoribonuclease T encoding *rnt* gene was identified in the 3 investigated organs and is involved in tRNA processing and thus in protein biosynthesis. It is the most important exoribonuclease in *E. coli* [23]. The 30S ribosomal protein S21 encoding *rpsU* gene was identified in the tonsils and is essential for proper function of the 30S ribosomal subunit during mRNA translation [24]. The genes encoding RNA polymerase subunit omega (*rpoZ*) and RNA polymerase sigma-54 factor (*rpoN*) were expressed in the ileocaecal lymph nodes. RpoZ restores denatured RNA polymerase *in vitro*, recruits the rest of the RNA polymerase core enzyme and is involved in *relA* gene expression. RelA plays a key role in the ‘stringent response’ due to amino acid and carbon starvation in *Salmonella* Typhimurium by the production of the ppGpp alarmone [25]. Since the stringent response in *Salmonella* Typhimurium and *Salmonella* Gallinarum is induced during infection of mice and chicks respectively [27], this stress response might also play a role during persistence of *Salmonella* Typhimurium in pigs. The *rpoN* encoded sigma factor (σ^54^) recognizes especially promoters of genes important for survival and adaptation under unfavourable conditions [26], [27].

### Surface structure and OMP assembly

Flagellin type 1 and type 2 encoding genes *fljB* and *fliC*, respectively, were identified from the tonsils. Flagellin is the main component of bacterial flagellae and only 1 of both genes is expressed at any given time in *Salmonella* Typhimurium, resulting in so called ‘phase variation’. It is generally believed that motility through flagellae mediates the initial interaction between bacterium and host. However, the contribution of flagellae to *Salmonella* Typhimurium virulence is strongly host-dependent. Flagellae contribute to invasion of the mucosa in the calf intestinal model [28], while neither flagellae, nor synthesis of the flagellar export machinery are necessary for pathogenicity in mice [29]. Nevertheless, a blocked *fljB* expression leads to attenuation of *Salmonella* Typhimurium *in vivo* in mice [30]. Furthermore, flagellin is required for the production of a variety of cytokines and inflammatory responses. No data about the role of flagellae in *Salmonella* Typhimurium persistence in pigs is available to date.

The gene encoding β-barrel integral protein BamA (*yaeT*) was expressed in the lymph nodes. The ‘β-barrel assembly machine’ or Bam anchors outer membrane proteins (OMPs), especially porins, into the outer membrane of Gram-negative bacteria. OMPs function as the interface between the bacterium and the environment and are therefore major virulence factors in these bacteria [31]. Expression of OMPs under iron limitation, oxidative stress and anaerobic conditions was shown for *Salmonella* Typhi [32]. The OMP AsmA was identified from the ileocaecal lymph nodes and was shown to be essential for *Salmonella* Typhimurium survival in *ex vivo* swine stomach contents [8]. A *Salmonella* Typhimurium Δ*asmA* is attenuated in virulence in mice, shows enhanced bile resistance and is defective in invasion of non-phagocytic HeLa cells. Therefore, the presence of AsmA in the outer membrane of *Salmonella* Typhimurium might be required for invasion in pigs [33].

### DNA replication and repair

The IVET screening allowed the identification of several genes that play a role in DNA replication and/or repair. As components of the primosome that creates RNA primers for DNA polymerase III on single stranded DNA, DnaC and DnaT are central proteins in DNA replication initiation. DnaT is involved in primosome assembly and DnaC associates with DnaB to form the DnaB-DnaC helicase complex that will unwind the DNA duplex prior to DNA replication [34]. *gyrB* encodes the B subunit of DNA gyrase, an essential enzyme in prokaryotic replication and transcription. DNA gyrase is a type II topoisomerase that unwinds the parental DNA duplex prior to replication and transcription [35]. It is a critical target for antimicrobial chemotherapy and it is therefore possible that *gyrB* is expressed as a response to gyrase inactivation by (unknown) host antimicrobial peptides. The ribonucleoside diphosphate reductase encoding *nrdB* is part of the *nrdAB* operon that is involved in DNA replication and repair by reducing ribonucleotides to deoxyribonucleotides. The *E. coli nrdA* promoter was found to respond strongly to DNA damaging chemicals and is suggested to function as a biosensor for DNA damage [36]. It is possible that this operon has a comparable function in *Salmonella* Typhimurium and that the operon is expressed upon DNA damage induced in the hostile porcine environment.

### Quorum sensing and anaerobic growth

Three genes were identified that play a role in anaerobic growth. The ‘fumarate nitrate reduction transcriptional regulator’ (Fnr) is a global regulator that plays a major role in bacterial switching from aerobic to anaerobic growth by regulation of a set of 311 known genes in *Salmonella* Typhimurium [37]. Fnr is only active in the absence of oxygen and among the 311 known genes under its regulation in *Salmonella* Typhimurium are reductases that use nitrate, nitrite and fumarate instead of oxygen as terminal electron acceptors [38]. Furthermore, Fnr is involved in regulation of *pflC* and *pflD*, encoding the ‘pyruvate formate lyase activase II’ and ‘putative formate acetyltransferase 2’ respectively, that were also identified from in IVET screening. Pyruvate formate lyase catalyzes the reversible reaction pyruvate + CoA ↔ acetyl−CoA + formate, a key reaction in the glucose fermentation route. It is likely that, due to the low oxygen tension in the ileocaecal lymph nodes, *Salmonella* Typhimurium is dependent on energy production routes that do not require oxygen for its survival in these organs.

One gene involved in *Salmonella* Typhimurium quorum sensing was identified from our screening. LsrR (previously YdeW) is a transcriptional repressor of the *lsr* operon that encodes a transport apparatus for auto-inducer 2 (AI-2) quorum sensing signals into the bacterial cell [39]. After entry in the cell, AI-2 is phosphorylated and will inhibit the LsrR repressor. The fact that *lsrR* is expressed in tonsils of pigs, might suggest that the *lsr* operon is of little importance for *Salmonella* Typhimurium persistence in porcine tonsils. In *Salmonella* Typhimurium, the *luxS* gene is directly involved in AI-2 production and deletion mutants lacking *luxS* are severely attenuated in SPI-1 gene expression. SPI-1 genes are of major importance for *Salmonella* Typhimurium invasion of epithelial cells in many animal hosts including pigs and do not play a role in persistence of porcine tonsils [5], [40], which might explain why the *lsr* operon repressor LsrR is expressed in the tonsils.

### Metabolism

Several genes were identified that have a known role in metabolism. *yadF* encodes a carbonic anhydrase, a zinc metalloenzyme that catalyzes the interconversion of CO_2_ and H_2_CO_3_. In *E. coli*, YadF becomes an essential enzyme for growth when the bacterium needs more H_2_CO_3_ [41] and the protein was already shown to induce virulence genes in *Vibrio cholerae* and several fungi [42]. Since *Salmonella* Typhimurium requires HCO_3_^−^ for the biosynthesis of pyrimidines, *yadF* expression might be induced due to a higher requirement of pyrimidines for survival in the host. As an ABC transporter, ArtP is part of the periplasmic arginine transport system encoded by the *artPIQMJ* operon, which transports arginine from the periplasmic space to the cytosol crossing the inner membrane [43]. All three mammalian forms of reactive oxygen species (ROS) are assumed to be formed by the same biochemical pathway that starts with the oxidation of L-arginine [44]. Since host produced ROS play a role in the protection against microbial pathogens like *Salmonella*, the arginine transport system might be expressed in *Salmonella* Typhimurium to deplete the arginine in the host environment, preventing the formation of antimicrobial ROS and thus playing a role in virulence. The lipopolysaccharide transport protein encoding *yhbG* (or *lptB*), expressed in the lymph nodes, is an ABC transporter located at the cytoplasmic side of the inner membrane and is essential for *E. coli* viability [45]. The Lpt machinery transports LPS from the cytosol to the outer membrane and is induced under stressful conditions when cell envelope integrity is affected. It is likely that the latter is the fact when *Salmonella* Typhimurium resides in the hostile porcine tissues and that expression of the Lpt machinery is essential to restore the cell envelope. Expression of *lysS*, encoding lysyl-tRNA-synthetase, was upregulated in the tonsils and couples L-lysine to free tRNA for protein synthesis. Since lysine is an essential amino acid for all animals, it is added to the feed of piglets. In contrast to pigs, *Salmonella* Typhimurium is able to produce lysine but also imports exogenous lysine via the lysine/cadaverine antiporter encoded by the *cad* locus, earlier shown to be involved in *E. coli* virulence. It is possible that upregulation of *lysS*, together with the exogenous lysine from the pig feed, plays a yet unknown role in *Salmonella* Typhimurium virulence in pigs. The gene encoding the ‘suppressor of copper sensitivity’ protein ScsA was expressed in the ileum. ScsA is a copper binding protein that possibly functions as a peroxidase, by preventing formation of free hydroxyl radicals resulting from the reaction of copper with hydrogen peroxide [46]. Copper sulphate has been extensively used as growth promoter in pig diets because of its selective antimicrobial effect on gut microflora and was also present in the feed that we administered to our pigs. Therefore, expression of the copper binding protein ScsA by *Salmonella* Typhimurium in the ileum of these pigs might protect the bacterium from the copper supplement's antimicrobial properties. As a member of the *kdg* regulon, the *kdgK* encoded ‘ketodeoxygluconokinase’ plays a role in the carbohydrate metabolism by phosphorylating 2-keto-3-deoxygluconate (KDG). The *citG2* gene encodes a ‘triphosphoribosyl-dephospho-CoA synthase’ that might be essential for the proper formation of the citrate lyase enzyme that catalyzes the cleavage of citrate to acetate and oxaloacetate, representing the initial step in all known bacterial citrate fermentation pathways [47]. Yet, no role in *Salmonella* virulence for these 2 genes has been established.

### Pathogenicity island genes

**Figure 1:**
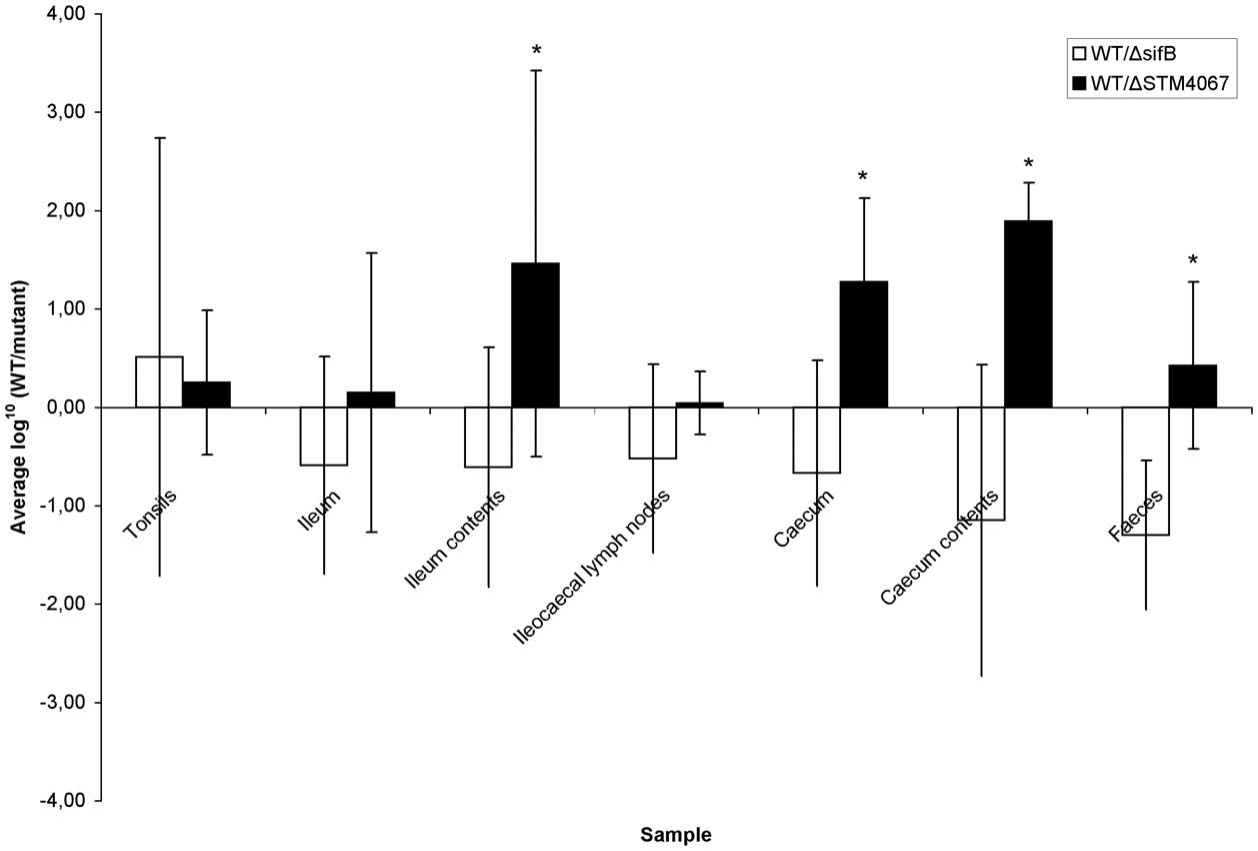
*Salmonella* Typhimurium wild type and Δ*sifB* or Δ*STM4067* colonization of pigs. Recovery of bacteria from various organs of 2 groups of 6 piglets orally inoculated with an equal mixture of the wild type *Salmonella* Typhimurium and Δ*sifB* or Δ*STM4067 Salmonella* respectively. The log^10^ value of the ratio of CFU per gram sample of the wild type and Δ*sifB* or Δ*STM4067* mutants is given as the mean ± standard deviation. A non-paramatric Mann-Whitney U test was performed to determine whether the log value of the WT/mutant ratios of the samples was significantly different from the log value of the WT/mutant ratios of the inocula. An asterisk (*) indicates that the output ratio was significantly different (p≤0.05) from that in the inoculum.

*Salmonella* Typhimurium possesses several pathogenicity islands (SPIs) that encode different virulence factors. Because many of these SPI-encoded genes are expressed on most laboratory media and we only identified IVET transformants that showed no *in vitro* activity (see Materials and Methods), it is not surprising that only 1 known SPI encoded gene was identified 3 weeks post inoculation of piglets. SifB is a SPI-2 effector, encoded outside the genomic SPI-2 region, that localizes to the *Salmonella* containing vacuole (SCV) and *Salmonella* induced filaments (Sifs) [48]. A *sifB* insertion mutant was found defective in colonization of calves and not chicks [49] and a *sifB* deletion mutant was not attenuated in mice after intraperitoneal inoculation. SPI-2 and its effectors are especially involved in intracellular survival and since we recently showed that *Salmonella* Typhimurium resides largely extracellularly in porcine tonsils [50], the upregulation of *sifB* in tonsils is rather unexpected. We compared the persistence capacity of *Salmonella* Typhimurium Δ*sifB*::kanR to that of the WT in a pig mixed infection experiment. Indeed, no significant differences in persistence capacity between the mutant and the WT could be detected in any of the examined samples (Figure 1). This is in accordance with [51], who found that deletion of the SPI-2 regulating *ssrA/B* operon did not significantly attenuate *Salmonella* Typhimurium for colonization in pigs compared to the wild type.

### Genes with putative and unknown function

Several *Salmonella* Typhimurium genes were identified with a putative or yet unknown function. YbjP is a ‘putative lipoprotein’ that is induced during the stationary phase and by acivicin and that is regulated by Lrp and RpoS [52]. Both regulators of *ybjP* play a known role in *Salmonella* Typhimurium virulence by regulation of various virulence genes and therefore it is possible that YbjP also exhibits a role in *Salmonella* Typhimurium virulence. For example, RpoS controls a regulon of genes required for protection against external stresses. Interestingly, Lrp was expressed in *Salmonella* Enteritidis after inoculation of chicks [13]. *yggE* encodes a ‘putative periplasmic immunogenic protein’ that is 2 to 3 times upregulated after UV-radiation and thermal stress in *E. coli*. Its promoter region has consensus sequences for RpoS and overexpression of RpoS results in enhanced *yggE* expression. YggE closely interacts with the cell membrane to maintain the cell's rigidity and intactness [53]. Yet, no function for YggE in *Salmonella* Typhimurium virulence is known, although [54] found *yggE* upregulated during *Salmonella* Typhimurium swarming and described the gene as a putative SPI-2 gene. The ‘putative ligase’ YgfA was expressed in the tonsils, ileum and lymph nodes in our IVET screening. Interestingly, the same gene was upregulated 3 weeks after *Salmonella* Enteritidis inoculation of chicks [13], suggesting a yet unknown role for *Salmonella* YgfA in persistence in different hosts. As expected from sequence homology to *E. coli*, via IVET identified *STM3020* encodes a putative LysR-type transcriptional regulator. The continuously increasing LysR family of transcriptional regulators is highly conserved among bacteria and is involved in a wide range of host-microbe interactions [55]. Possibly, the *STM3020* gene product is a yet unspecified transcriptional regulator involved in *Salmonella* Typhimurium colonization of and persistence in pigs. In the lymph nodes, the *citX2* gene was expressed encoding a ‘putative cytoplasmic protein’ with a yet unknown function In *Salmonella* Typhimurium. *STM4067* is part of the *STM4065-4066-4067* operon of which STM4066 has aminoimidazole riboside (AIR) kinase activity, phosphorylating AIRs and satisfying the thiamine requirement of *pur* mutant strains [56]. Because our IVET library was constructed in a Δ*purA Salmonella* Typhimurium, which lacks the enzyme adenylosuccinate synthase, required for synthesis of adenosine 5′-monophosphate (AMP). For that reason, a bias to the upregulation and subsequent identification of genes involved in AMP synthesis, or more generally the purine biosynthesis pathway of *Salmonella* Typhimurium, from the IVET *in vivo* screening might be expected. To rule out this bias, the *Salmonella* Typhimurium Δ*STM4067*::kanR was tested in a mixed infection experiment with the WT. The Δ*STM4067*::kanR was attenuated in the ileum and ileum contents, the caecum and caecum contents and the faeces and these differences were significant in the ileum contents (*p* = 0.04), the caecum (*p* = 0.033) and caecum contents (*p* = 0.033) and the faeces (*p* = 0.032; Figure 1). The fact that Δ*STM4067*::kanR was attenuated in the pigs' intestines compared to the WT proved that our IVET selection actually resulted in the identification of *Salmonella* genes involved in persistence in pigs.

### *Salmonella* Typhimurium specifically expresses *rpoZ* and *efp* in porcine lymph nodes

The average OD_650_ between the separate IVET transformant 16 h cultures did not significantly differ (*p*>0.05), so each transformant was equally present in the final inoculum and, consequently, had an equal chance to establish an infection in pigs.

First, we compared the *Salmonella* Typhimurium gene expression pattern between tonsils and ileocaecal lymph nodes. The majority of the identified genes showed no tissue-specific expression pattern (*p*>0.05) and we were unable to find genes specifically expressed in the tonsils, although *artP*/*ybjP* was only isolated from the tonsils (and not from the lymph nodes or ileum) with a frequency of 2/6 (*p* = 0.227).

**Figure 2:**
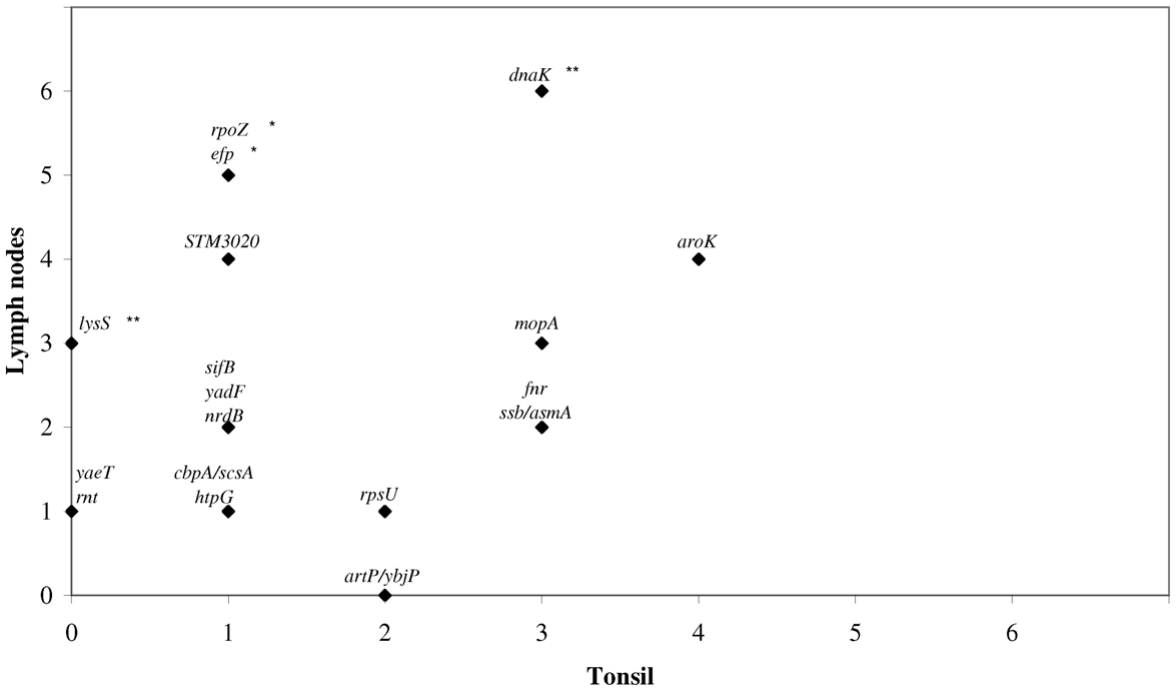
*Salmonella* Typhimurium genes differentially expressed in porcine tonsils and lymph nodes. The frequency of *Salmonella* Typhimurium gene expression in the tonsils and/or ileocaecal lymph nodes of 6 pigs, 20 days post inoculation with approximately 1×10^9^ CFU of an IVET pool containing 32 transformants that were identified in an initial IVET screening. Genes with significantly different isolation frequencies in tonsils and lymph nodes (p≤0.05) are indicated with an asterisk (*). Genes with borderline different isolation frequencies in tonsils and lymph nodes (0.05<*p*≤0.10) are indicated with two asterisks (**).

However, *efp*, encoding the elongation factor P, and *rpoZ*, encoding the RNA polymerase omega subunit, were expressed in the lymph nodes of 5/6 pigs and in the tonsils of 1/6 pigs, and these frequencies were significantly different (*p* = 0.04). It can thus be assumed that both genes are specifically expressed in ileocaecal lymph nodes during *Salmonella* Typhimurium persistence in pigs. *STM3020* was expressed in 4 of 6 lymph nodes and in 1 of 6 tonsils (*p* = 0.121). The comparison of isolation frequency of transformants between tonsils and lymph nodes is graphically presented in Figure 2.

The results of the ileum could not be statistically analyzed, because too few colonies were isolated at direct plating (as expected in the used infection model), and because enrichment of samples might bias the result of a sample towards 1 specific transformant. However, interestingly, the genes *efp*, *rpoZ* and *STM3020* are also expressed at higher frequencies in the ileum than in the tonsils: 4/6, 3/6 and 2/6, respectively. It is possible that expression of these genes is required for colonization of the ileum and subsequent penetration of and persistence in the underlying lymphoid tissues, suggesting that *Salmonella* Typhimurium exploits different genes for tonsil and intestinal colonization, as proposed earlier [5].

The chaperone Hsp60 encoding gene *dnaK* was expressed in the lymph nodes of all 6 piglets and in 3 of 6 examined tonsils and ileum samples and this difference in frequency was borderline significant (*p* = 0.091). In addition to this, the shikimate kinase encoding *aroK* was isolated at equal frequencies from both the tonsils and the lymph nodes (4/6) and was also expressed in 3/6 ileum samples. These results suggest a general role for both genes in *Salmonella* persistence of porcine tissues.

The lysyl tRNA synthetase encoding *lysS* was expressed in 3 of 6 lymph nodes and not in the tonsils (*p* = 0.091). Interestingly, after modification onto highly-conserved lysine residues by the enzymes YjeK and PoxA, of which the latter is a paralog of lysyl tRNA synthetase, *efp* was shown to contribute to *Salmonella enterica* virulence in a mouse model [57]. The expression of *lysS* and *efp* suggests that the family of lysyl tRNA synthetases, and the proteins that they modify, might be involved in *Salmonella* virulence in porcine lymph nodes, by regulating the synthesis and/or activation of a limited subset of proteins. Indeed, recently a role for *Salmonella* Typhimurium *poxA* in colonization of porcine tissues was established, providing additional evidence for an important role for lysyl tRNA synthetases in *Salmonella* pathogenesis in pigs [58].

Eighteen different IVET transformants, corresponding to 21 different gene fragments, were isolated from the 3 examined organs (see Supporting Information S2 for an overview per organ). Among others, the gene encoding STM4067, that we identified as a factor involved in *Salmonella* persistence in the intestines of pigs, could not be recovered from the reintroduction experiment. This shows that the ‘bottleneck’ in the original IVET screening was to a certain extent but not completely circumvented in the reintroduction experiment. Furthermore, isolation of low numbers of bacteria and, consequently, an increased variation between pigs are inherent to the infection model we use to study *Salmonella* persistence in pigs, compared to invasion and colonization studies in the early phase of *Salmonella* infection [59]. So there is a reasonable chance of missing several IVET transformants, even when pigs are inoculated with a limited pool.

Our results show that conclusions about tissue-specific gene expression patterns from an initial IVET screening must be drawn very carefully. When the lymph-node specific expressed genes *rpoZ* and *efp* are considered, *rpoZ* was identified from only the ileocaecal lymph nodes in the initial IVET screening. However, *efp* was identified from the lymph nodes and from the tonsils in the initial screening, but remained absent from the latter in the subsequent reintroduction experiment. In contrast, *lysS* that was expressed only in the tonsils in the initial screening, was expressed in 3 lymph nodes but not in the tonsils in the second IVET experiment. Furthermore, *aroK* and *dnaK* that are probably involved in overall *Salmonella* persistence in pigs, were only expressed in porcine lymph nodes in the initial screening (Figure 2 and Table 2).

In general, our results emphasize that for proper interpretation of tissue-specific gene expression patterns using IVET screening, a subsequent infection of the host animal that is subject of study with a limited number of IVET transformants is required, to minimize bottleneck effects and to get an indication of tissue-specificity.

### Conclusion

Using the IVET screening, we were able to identify 37 *Salmonella* Typhimurium genes that were expressed 3 weeks post inoculation of pigs. To our knowledge, this study is the first to identify genes that might play a role in *Salmonella* Typhimurium persistence in pigs. After *Salmonella* entry, the porcine immune system will fight the bacterium by establishing a hostile environment. It can thus be expected that the expression of *Salmonella* genes encoding proteins that protect the bacterium against stressful conditions like antimicrobial proteins, cell wall degradation, lack of oxygen, etc. is upregulated during *Salmonella* Typhimurium persistence in pigs. Interestingly, there is little or no overlap between the genes expressed in our study and the genes previously identified by an IVET [10] and STM [9] approach, respectively 2 and 3 days post oral inoculation of pigs with *Salmonella* Typhimurium. This finding indicates that different sets of *Salmonella* genes could be involved in colonization (2–3 days pi) of and persistence (20 days pi) in pigs.

A possibility existed that the total number of different transformants exceeded a bottleneck above which each pool member no longer had an equal chance of establishing itself within the IVET population. To minimize these bottleneck effects and to find tissue-specific gene expression patterns, we reintroduced the pool of different IVET transformants identified in the initial IVET screening in 6 *Salmonella*-free piglets. We found that *efp* and *rpoZ* were specifically expressed in porcine lymph nodes during *Salmonella* persistence in pigs. Genes that were expressed at higher frequencies in the lymph nodes than in the tonsils, also appeared to be more frequently expressed in the ileum, suggesting that comparable sets of *Salmonella* genes are involved in colonization of the ileum and lymph nodes. Furthermore, *dnaK* and *aroK* were found to play a general role in *Salmonella* persistence in pigs. Although the results obtained from the IVET reintroduction experiment give a more reliable indication of tissue-specific *Salmonella* gene expression patterns than the initial screening, extrapolation of such expression patterns should still be drawn very carefully, among others due to the limited number of piglets used.

It was also possible that the inoculation dose applied in our initial screening experiment was insufficient for some of the IVET transformants to initiate infection in pigs. To assess a role in *Salmonella* pathogenicity for IVET identified genes, we constructed *Salmonella* substitution mutants for 2 genes that were expressed in the initial IVET screening. We identified STM4067 in a subsequent mixed infection experiment as a factor for intestinal *Salmonella* Typhimurium persistence in pigs. Finally, we must emphasize that although the IVET identified genes are expressed during *Salmonella* Typhimurium persistence in pigs, these genes are not per se essential for persistence, as becomes clear from the mixed infection experiment with the WT and the Δ*sifB*::kanR.

## Supporting Information

Supporting Information S1 **Primers used in this study.** Primers used in this study to create the *Salmonella* Typhimurium Δ*purA*, Δ*sifB* and Δ*STM4067* substitution mutants and Y-linker component sequences and *purA*- and ‘Y-linker’-primer sequences used for sequencing IVET fusion strains.
(DOC)

Supporting Information S2 **Overview of identified IVET transformants per organ.** Identification of IVET transformants recovered from the tonsils, ileocaecal lymph nodes and ileum in the reintroduction experiment, in which 6 piglets were orally inoculated with approximately 5×10^8^ CFU of a pool of 32 IVET transformants.
(DOC)
