# Supplementary material for: Tissue-Specific Salmonella Typhimurium Gene Expression during Persistence in Pigs
Source: PLoS One. 2011 Aug 24;6(8):e24120. doi: 10.1371/journal.pone.0024120 (PMC3161100; doi:10.1371/journal.pone.0024120)
Supplement: Supporting Information S1 — Primers used in this study. Primers used in this study to create the Salmonella Typhimurium ΔpurA, ΔsifB and ΔSTM4067 substitution mutants and Y-linker component sequences and purA- and ‘Y-linker’-primer sequences used for sequencing IVET fusion strains. (DOC) [file pone.0024120.s001.doc]

| Primers | Sequences |
| --- | --- |
| *purA* Forward | 5’-TGAAAAAGCGATGGTAGAATCCATTTTTAAGCAAACGGTGATTTTGAAAAT GTG TAGGCTGGAGCTGCTTC-3’ |
| *purA* Reverse | 5’-GTATCACACTGTTAGCCCGGCAAGCAAAATATCTGCCAGGCGTACCAGATA TATGAATATCCTCCTTAG -3’ |
| *sifB* Forward | 5’-ACCACCTATTCCAGTAATGAAGTATCATATAATCACTTGTGGTCTACATTTG  TG TAGGCTGGAGCTGCTTC-3’ |
| *sifB* Reverse | 5’-TGGTTTTGGTATTGCCAGGGGATTGTAAATCCATACTATTTATGGTGTGACA  TATGAATATCCTCCTTAG-3’ |
| *STM4067* Forward | 5’-TTTCCAGCCATTCGCTGGCACAAGCCATGACCGTGAAATAAGGAGTCATCT  GTGTAGGCTGGAGCTGCTTC-3’ |
| *STM4067* Reverse | 5’-ACGTCTTCGCCAGCAGGGGACGTTTTACCGTCCCCGGCAGGGTGAAGTCGC  ATATGAATATCCTCCTTAG-3’ |
| Linker 1 | 5’-TTTCTGCTCGAATTCAAGCTTCTAACGATGTACGGGGACACATG-3’ |
| Linker 2 | 5’-TGTCCCCGTACATCGTTAGAACTACTCGTACCATCCACAT-3’ |
| purA primer | 5’-TTGATTACGAGAGTATGGCCT-3’ |
| Y-linker primer | 5’-CTGCTCGAATTCAAGCTTCT-3’ |
