# Supplementary material for: Tissue-Specific Salmonella Typhimurium Gene Expression during Persistence in Pigs
Source: PLoS One. 2011 Aug 24;6(8):e24120. doi: 10.1371/journal.pone.0024120 (PMC3161100; doi:10.1371/journal.pone.0024120)
Supplement: Supporting Information S2 — Overview of identified IVET transformants per organ. Identification of IVET transformants recovered from the tonsils, ileocaecal lymph nodes and ileum in the reintroduction experiment, in which 6 piglets were orally inoculated with approximately 5×10^8^ CFU of a pool of 32 IVET transformants. (DOC) [file pone.0024120.s002.doc]

| Tonsil | | | | | |
| --- | --- | --- | --- | --- | --- |
| 1 | 2 | 3 | 4 | 5 | 6 |
| dnaK | aroK | ssb/asmA | rpsU | fnr | dnaK |
| efp | aroK | dnaK | rpsU | sifB | nrdB |
| dnaK | aroK | fnr | rpsU | sifB | htpG |
| purA | aroK | mopA | rpsU | aroK | dnaK |
| dnaK | aroK | dnaK | rpsU | sifB | htpG |
| dnaK | aroK | dnaK | rpsU | sifB | dnaK |
| rpsU | aroK | dnaK | rpsU | STM3020 | nrdB |
| fnr | aroK | dnaK | mopA | mopA | nrdB |
| rnt | aroK | mopA | rpsU | fnr | dnaK |
| cbpA/scsA | aroK | purA | rpsU | STM3020 | dnaK |
| efp | aroK | dnaK | rpsU | STM3020 | htpG |
| efp | aroK | ssb/asmA | rpsU | aroK | dnaK |
| purA | aroK | dnaK | rpsU | fnr | htpG |
| purA | aroK | ssb/asmA | mopA | sifB | htpG |
| ssb/asmA | aroK | dnaK | rpsU | STM3020 | htpG |
| yjeT/purA | aroK | mopA | rpsU | yadF | htpG |
| fnr | aroK | dnaK | rpsU | STM3020 | dnaK |
| artP/ybjP | aroK | mopA | rpsU | aroK | htpG |
| dnaK | aroK | mopA | rpsU |  | aroK |
| dnaK | aroK | dnaK | rpsU |  | htpG |
| aroK | aroK | dnaK | rpsU |  | dnaK |
| dnaK | aroK | dnaK | purA |  | dnaC/dnaT |
| rpoZ | aroK | mopA | rpsU |  | artP/ybjP |

| Ileocaecal lymph nodes | | | | | |
| --- | --- | --- | --- | --- | --- |
| 1 | 2 | 3 | 4 | 5 | 6 |
| rpoZ | dnaK | aroK | yaeT | efp | sifB |
| rpoZ | aroK | yadF | rpsU | dnaK | efp |
| mopA | efp | rpoZ | yaeT | sifB | efp |
| mopA | STM3020 | rpoZ | rpsU | rpoZ | htpG |
| efp | efp | rpoZ | aroK | rpoZ | sifB |
| efp | aroK | dnaK | rpsU | ssb/asmA | rpoZ |
| efp | dnaK | mopA | yaeT | dnaK | rpoZ |
| lysS | efp | fnr | yaeT | sifB | htpG |
| nrdB | fnr | mopA | yaeT | dnaK | efp |
| aroK | fnr | rpoZ | yaeT | dnaK | htpG |
| rpoZ | nrdB | mopA | yaeT | efp | htpG |
| lysS | dnaK | mopA | yaeT | rpoZ | efp |
| yadF | dnaK | rpoZ | rpsU | dnaK | STM3020 |
| cbpA/scsA | efp | lysS | yaeT | lysS | rpoZ |
| dnaK | efp | rnt | dnaK | lysS | efp |
| aroK | aroK | lysS | dnaK | efp | rpoZ |
| STM3020 | aroK | aroK | yaeT | fnr | rpoZ |
| rpoZ | dnaK | mopA | yaeT | ssb/asmA | dnaK |
| mopA | efp | mopA | rpsU | ssb/asmA | htpG |
| yadF | rpoZ | STM3020 | dnaK | sifB | efp |
| ssb/asmA | aroK | cbpA/scsA |  | rpoZ | efp |
| aroK | nrdB | mopA |  | lysS | rpoZ |
| aroK | nrdB | rpoZ |  | efp | rpoZ |

| Ileum | | | | | |
| --- | --- | --- | --- | --- | --- |
| 1 | 2 | 3 | 4 | 5 | 6 |
| yadF | efp | dnaK | mopA | efp | STM3020 |
| aroK | efp | dnaK | dnaK | rnt | STM3020 |
| rpsU | efp | dnaK | mopA | rpoZ | STM3020 |
| dnaT | efp | dnaK | mopA | aroK | STM3020 |
| rpoZ | efp | dnaK | efp | aroK | STM3020 |
| ssb/asmA | efp | dnaK | aroK | aroK | STM3020 |
| lysS | yadF | dnaK | aroK | dnaC/dnaT | STM3020 |
| rnt | efp | dnaK | aroK | rnt | STM3020 |
| STM3020 | efp | dnaK | efp | efp | STM3020 |
| STM3020 | ssb/asmA | dnaK | dnaK | dnaK | STM3020 |
| efp |  |  | aroK | aroK |  |
| efp |  |  | mopA | aroK |  |
| efp |  |  | aroK | rnt |  |
| efp |  |  | rpoZ | aroK |  |
| efp |  |  | dnaK | aroK |  |
|  |  |  | aroK | efp |  |
|  |  |  | efp | efp |  |
|  |  |  | aroK | cbpA/scsA |  |
|  |  |  | aroK | aroK |  |
|  |  |  | rnt |  |  |
